# Perceived xerostomia, stress and periodontal status impact on elderly oral health-related quality of life: findings from a cross-sectional survey

**DOI:** 10.1186/s12903-020-01183-7

**Published:** 2020-07-10

**Authors:** João Botelho, Vanessa Machado, Luís Proença, Maria João Oliveira, Maria Alzira Cavacas, Luís Amaro, Artur Águas, José João Mendes

**Affiliations:** 1 Periodontology Department, Clinical Research Unit (CRU), Centro de Investigação Interdisciplinar Egas Moniz (CiiEM), Instituto Universitário Egas Moniz, Egas Moniz Cooperativa de Ensino Superior Campus Universitário, Quinta da Granja, 2829 - 511 Almada, Portugal; 2 Clinical Research Unit (CRU), Centro de Investigação Interdisciplinar Egas Moniz (CiiEM), Egas Moniz, CRL, Quinta da Granja, Almada, 2829 - 511 Portugal; 3 Quantitative Methods for Health Research (MQIS), CiiEM, Egas Moniz, CRL, Quinta da Granja, Almada, 2829 - 511 Portugal; 4 Department of Anatomy and Unit for Multidisciplinary Research in Biomedicine (UMIB), Institute of Biomedical Sciences Abel Salazar (ICBAS), University of Porto, Jorge de Viterbo Ferreira, 228, Porto, 4050-313 Portugal; 5 Health Centers grouping (HCG) Almada-Seixal, Regional Health Administration of Lisbon and Tagus Valley (RHALTV), Av. Estados Unidos da América 77, Lisbon, 1700-179 Portugal

**Keywords:** Xerostomia, Stress, Elders, Oral health-related quality of life, Periodontitis, Tooth loss, Aging

## Abstract

**Background:**

To investigate if self-perceived xerostomia and stress are significant variables on the Oral-Health Related Quality of Life (OHRQoL) of elderly patients, considering the periodontal status, oral hygiene habits and sociodemographic characteristics simultaneously.

**Methods:**

The study cohort included 592 participants (320 females/272 Males), aged 65 years or older, representing the elder inhabitants of the Study of Periodontal Health in Almada-Seixal (SoPHiAS). Patients answered a socio-demographic and oral hygiene habits questionnaire. The Oral Health Impact Profile-14 (OHIP-14), Summated Xerostomia Inventory-5 (SXI-5) and Perceived Stress Scale-10 (PSS-10) were used. Full-mouth circumferential periodontal inspection was carried out. Multivariable regression analyses were used considering the level of periodontitis, clinical characteristics, the number of teeth, SXI, PSS-10, age, gender and oral hygiene habits.

**Results:**

Self-perceived xerostomia and stress showed a positive significant correlation with OHRQoL and each of its domains. Multiple linear regression analysis demonstrated the significant impact of SXI-5 (B = 1.20, *p* <  0.001) and PSS-10 (B = 0.35, *p* <  0.001) on the OHRQoL. SXI-5 (Odds Ratio (OR) = 1.28, *p* <  0.001) and PSS-10 (OR = 1.03, *p* = 0.022) were associated with a more frequently affected OHRQoL. The number of missing teeth, being male, mean probing depth and mean clinical attachment loss were also significant towards a frequently affected OHRQoL. Conversely, age was negatively associated with a lower OHRQoL.

**Conclusion:**

Self-perceived xerostomia and stress are significant variables towards OHRQoL in elderly patients. Future studies should consider these self-perceived xerostomia and stress when investigating the impact of periodontitis and missing teeth on quality of life of older adults.

## Background

Patient-reported outcomes (PROs) are emerging key measures to aid oral health care decision-making policies [1]. In a disease or treatment situation, patients’ perspective is often the most significant result over clinical outcomes [2]. Besides, in a world with a population ageing so fast, doing so healthy and with good quality of life has become an exciting matter to study [3].

Periodontal diseases are highly prevalent among older people and remain a substantial epidemiological challenge [4–6]. Periodontal diseases negatively impact on oral health-related quality of life (OHRQoL), especially with the worsening and extent of disease, and with special relevance in the elderly populations [7–11]. Notwithstanding, the lost quality of life can be recovered after nonsurgical periodontal therapy [12].

The impact of periodontitis on the OHRQoL, in elder populations, is poorly studied. Nevertheless, though periodontitis leads to poorer quality of life, its clinical consequences as missing teeth and denture use have apparently a higher impact [13, 14]. Nevertheless, it is important to investigate whether other confounders could influence the quality of life perception. Stress has been linked to both periodontitis and OHRQoL [15, 16], and xerostomia has been associated to poorer quality of life [17, 18]. Self-reported objective and subjective dry mouth complaints impact OHRQoL in senior men and women, because the majority has medical conditions and medications that might cause xerostomia [19, 20]. Besides, perceived stress has also been linked to worse OHRQoL in older people [21]. However, the impact of self-perceived xerostomia and stress together with periodontitis on the OHRQoL of elderly individuals has never been explored, and may be potential influential variables.

Therefore, we aimed to evaluate whether self-perceived xerostomia and stress could influence OHRQoL in a representative elderly population, considering also the extent of periodontitis, the number of missing teeth, clinical variables and oral hygiene habits.

## Methods

### Ethics and study design

The Study of Periodontal Health in Almada-Seixal (SoPHiAS) is a population-based representative study, with a target population living in the municipalities of Almada and Seixal (Portugal) [22]. This study was approved by the Research Ethics Committee of the Regional Health Administration of Lisbon and Tagus Valley, IP (Portugal) (8696/CES/2018) and in accordance with the Declaration of Helsinki, as revised in 2013. Participants were informed about their periodontal status after examination. Patients diagnosed with periodontal disease were referred to the Egas Moniz Dental Clinic (EMDC) for treatment without additional costs [22]. The study followed the STrengthening the Reporting of OBservational studies in Epidemiology (STROBE) guidelines [23].

### Sample size and measurement reproducibility

A fully detailed report on elderly sampling strategy and measurement reproducibility are mentioned in [22]. A total of 1064 participants, aged 18 to 95 years, gave their consent and were examined [22]. For the purpose of this study, a subset of 592 participants, 320 women and 272 men, with 65 years old or over were studied.

### Periodontal examination and clinical variables

Each clinical examination was performed using proper lightening with the individuals seated on an adjustable stretcher in the FHU’s medical office. Periodontal examination was made as described in Botelho et al. [22]. Periodontitis case definitions were defined according to the new AAP/EFP consensus [24].

### Questionnaires

Information on sociodemographic characteristics and behaviours was collected by a self-reported questionnaire. The questionnaire covered questions on the following items: 1) gender, age, marital status, educational level, occupation; 2) monthly family gross income; 3) smoking habits; 4) oral hygiene-related behaviours (tooth brushing frequency, interproximal cleaning); 5) attitudes and awareness towards oral health.

Participants completed the Portuguese versions of the Oral Health Impact Profile-14 (OHIP-14-PT) [25] to assess OHRQoL, Summated Xerostomia Inventory-5 (SXI-5-PT) [26] to quantify xerostomia and Perceived Stress Scale-10 (PSS-10-PT) to estimate recognised stress [27].

The OHIP-14 consists of 14 questions representing seven domains (functional limitation, physical pain, psychological discomfort, physical disability, psychological disability, social disability and handicap) of OHRQoL. Each question is scored by 0 (never,) 1 (hardly ever), 2 (occasionally), 3 (fairly often) and 4 (very often). Thus, a higher score indicates poorer OHRQoL. Each pair of questions represents one of seven domains of the OHIP-14. The sum of the scores of the 14 questions ranges from 0 to 56 and the sum of each domain ranges from 0 to 8 (Slade et al. 1997). Further, individuals were categorised as frequently affected individual with respect to OHRQoL (answering with 3 or 4 to at least one of the questions in the OHIP-14) or with less affected individuals (responding with 0, 1 or 2 on all the items) [13].

The SXI-5 is a 5-questions tool where each question is scored by 0 = never, 1 = occasionally 2 = frequently. The scores from the five questions are summed, with the result representing the degree of xerostomia the subject feels [26].

The PSS-10 is 10-items instrument indicated to assess self-perceived stress. Each of the items on the PSS-10 is rated on a 5-point Likert scale, and each question is scored 0 = never, 1 = almost ever, 2 = sometimes, 3 = fairly often and 4 = very often. The PSS-10 consists of two domains: six positively (items 1, 2, 3, 6, 9 and 10) and four negatively (items 4, 5, 7 and 8, that require reversion) worded items. Total scores range from 0 to 40, with higher scores indicating higher levels of perceived stress [28].

### Assessment of confounders

Furthermore, the participants have also been categorised into three groups according to the extent of periodontitis: no disease, localized periodontitis and generalized periodontitis [24]. Concerning oral hygiene, patients were categorized for their interproximal hygiene (no = 0, occasionally = 1, and yes = 2) and for frequency of toothbrushing per day (less than one time per day = 0, one time per day = 1, and two or more times per day = 2). Also, patients were questioned to the use of dentures and registered as a dichotomous variable (no = 0, or yes =1).

### Statistical methods

The total scores of OHIP-14, PSS-10 and SXI-5 were calculated and their correspondent descriptive measures (mean and standard deviation (SD)) were computed. For analysis purposes, these scores were considered as continuous variables. The data analyses were conducted for all participants and for sample subsets, according to gender and periodontitis extent. Mann-Whitney and Kruskal-Wallis tests were used to compare OHRQoL scores as a function of gender and periodontitis extent. For categorical variables, the analyses were performed using Chi-square test. Spearman’s rank correlation coefficient (rho) was used to analyse the correlation of OHIP-14 scores with PSS-10 and SXI-5 total scores, number of missing teeth and periodontal clinical variables. The effect size of correlations was analysed according to Cohen’s standard. Further, a multiple forward stepwise linear regression analysis was carried out in order to evaluate the impact of those variables on the OHIP-14 total score. Next, a multivariable forward stepwise logistic regression was applied using the dichotomised dependent OHIP-14 variable “frequently affected” vs “less affected” OHRQoL as in [13]. Odds ratio (OR) and correspondent 95% confidence level intervals (95% CI) were calculated. Data were analysed using IBM SPSS Statistics, v. 25, (NY, USA). A level of significance of 5% was considered in all inferential analyses.

## Results

### Sample description

In general, the average total OHIP-14 score indicates that these participants perceived their OHRQoL as modest, although men perceived better OHRQoL than women (Table 1). Regarding periodontitis extent, 70.9% had periodontitis with 24.0 and 47.0% being localized and generalized forms, respectively. Men had more prevalence of localized and generalized periodontitis than women. Women had significantly more missing teeth, although self-reporting better oral hygiene habits than men, on interproximal cleaning and toothbrushing. Almost half of the population were denture wearers, with the majority using partial and removable acrylic types. Besides, this population self-reported moderate signs of dry-mouth and stress with significant differences between gender, with men experiencing more stress and women more signs of xerostomia.

**Table 1:** Oral Health Impact Profile (OHIP-14), Summated Xerostomia Inventory-5 (SXI-5) and Perceived Stress Scale-10 (PSS-10) scores, age, number of missing teeth, periodontitis extent, oral hygiene variables and denture wearers, according to gender and for the overall participants (*N* = 592)

	Women (*n* = 320)	Men (*n* = 272)	*P*-value	Overall (*N* = 592)
**OHIP-14 Total, mean (SD)**	9.57 (11.70)	5.83 (8.54)	< 0.001*	7.85 (10.53)
**OHIP-14 domains, mean (SD)**				
Functional limitation	1.23 (2.01)	0.85 (1.58)	0.032*	1.05 (1.83)
Physical pain	2.54 (2.70)	1.93 (2.40)	0.008*	2.26 (2.58)
Psychological discomfort	1.67 (2.58)	0.86 (1.94)	< 0.001*	1.30 (2.34)
Physical disability	1.58 (2.47)	0.97 (1.91)	0.003*	1.30 (2.25)
Psychological disability	1.28 (2.20)	0.58 (1.54)	< 0.001*	0.96 (1.95)
Social disability	0.41 (1.27)	0.17 (0.74)	0.029*	0.30 (1.07)
Handicap	0.87 (1.87)	0.47 (1.34)	0.008*	0.68 (1.66)
**SXI-5, mean (SD)**	7.1 (2.1)	6.3 (1.5)	0.001*	6.7 (1.9)
**PSS-10, mean (SD)**	14.2 (8.2)	16.1 (7.6)	0.002*	15.1 (8.0)
**Age, mean (SD)**	71.9 (6.2)	73.4 (6.6)	0.007*	72.6 (6.4)
**Mean PD (mm), mean (SD)**	1.88 (0.70)	2.00 (0.88)	0.372*	1.94 (0.79)
**Mean CAL (mm), mean (SD)**	2.86 (1.28)	3.22 (1.76)	0.259*	3.02 (1.53)
**Mean Rec (mm), mean (SD)**	0.98 (0.94)	1.24 (1.26)	0.047*	1.10 (1.10)
**Mean BoP (%), mean (SD)**	15.1 (20.7)	14.9 (21.5)	0.026*	15.0 (21.1)
**Missing teeth, mean (SD)**	10.9 (6.7)	11.9 (6.8)	< 0.001*	12,72 (6.77)
**Periodontitis, n (%)**				
**Severity**				
Healthy	104 (32.5)	68 (25.0)	**0.032**^**#**^	172 (29.1)
Stage I (Mild)	48 (15.0)	36 (13.2)		84 (14.2)
Stage II (Moderate)	79 (24.7)	77 (28.3)		156 (26.4)
Stage III (Severe/Advanced)	89 (27.8)	91 (33.5)		180 (30.4)
**Extent**				
No	104 (32.5)	68 (25.0)	0.135^#^	172 (29.1)
Localized	73 (22.8)	69 (25.4)		142 (24.0)
Generalized	143 (44.7)	135 (49.6)		278 (47.0)
**Interproximal cleaning, n (%)**				
No	227 (70.9)	233 (85.7)	**< 0.001**^#^	460 (77.7)
Occasionally	35 (10.9)	18 (6.6)		53 (9.0)
Yes	58 (18.1)	21 (7.7)		79 (13.3)
**Toothbrushing frequency per day, n (%)**				
0	8 (2.5)	18 (6.6)	**< 0.001**^#^	26 (4.4)
1	79 (24.7)	114 (41.9)		193 (32.6)
2+	233 (72.8)	140 (51.5)		373 (63.0)
**Denture wearer, n (%)**				
No	137 (42.8)	170 (62.5)	**< 0.001**^#^	307 (51.9)
Yes	183 (57.2)	102 (37.5)		285 (48.1)
**Denture extent, n (%) (n = 285)**				
Partial	128 (69.9)	77 (75.5)	–	205 (71.9)
Full	12 (6.6)	5 (4.9)		17 (6.0)
Both	43 (23.5)	20 (19.6)		63 (22.1)
**Type of denture, n (%) (n = 285)**				
Removable Acrylic	124 (67.8)	73 (71.6)	**–**	197 (69.1)
Removable Metallic	58 (31.7)	24 (23.5)		82 (28.8)
Removable Acrylic and Metallic	1 (0.5)	0 (0)		1 (0.4)
Fixed	1 (0.5)	5 (4.9)		6 (2.1)

*Mann-Whitney test. ^#^Chi-square test. Significant differences identified in bold (*p* < 0.05)

*Mean BoP* Mean bleeding of probing, *Mean CAL* Mean clinical attachment loss, *Mean PD* Mean probing depth; Mean Rec – Mean gingival recession; OHIP-14 - Oral Health Impact Profile-14; PSS-10 - Perceived Stress Scale-10; SD – standard deviation; SXI-5 - Summated Xerostomia Inventory-5

Furthermore, in the overall sample, patients with generalized periodontitis stated average similar OHRQoL than patients with no periodontitis and localized (Table 2). Only the handicap domain demonstrated notable differences in men, between no disease patients and both localized and generalized periodontitis inmates. Periodontitis severity showed to influence OHRQoL levels, with more severe cases presenting worse OHRQoL parameters (Table 3). Also, in this analysis, only the functional limitation domain had differences among women between no disease and the different types of periodontitis.

**Table 2:** Oral Health Impact Profile (OHIP-14) scores, total and for each domain, presented as mean and standard deviation (SD), according to gender and periodontitis extent

	Women (***n*** = 320)				Men (***n*** = 272)				Overall (***N*** = 592)			
	ND	L	G	***P***-value #	ND	L	G	***P***-value #	ND	L	G	***P***-value #
**OHIP-14, mean (SD)**	9.02 (11.90)	9.29 (10.45)	10.11 (12.23)	0.430	4.28 (6.46)	5.64 (8.78)	6.71 (9.24)	0.165	7.15 (10.35)	7.51 (9.81)	8.42 (10.98)	0.178
**OHIP-14 domain, mean (SD)**												
Functional limitation	1.19 (2.23)	1.25 (1.70)	1.25 (2.00)	0.435	0.71 (1.39)	0.77 1.37)	0.96 (1.76)	0.732	1.00 (1.95)	1.01 (1.56)	1.11 (1.89)	0.466
Physical pain	2.44 (2.68)	2.56 (2.45)	2.61 (2.85)	0.897	1.81 (2.32)	1.77 (2.44)	2.07 (2.43)	0.501	2.19 (2.56)	2.18 (2.47)	2.35 (2.66)	0.866
Psychological discomfort	1.56 (2.60)	1.75 (2.45)	1.66 (2.61)	0.405	0.53 (1.69)	0.84 (1.91)	1.04 (2.07)	0.079	1.15 (2.33)	1.31 (2.24)	1.36 (2.38)	0.178
Physical disability	1.57 (2.47)	1.36 (2.18)	1.68 (2.62)	0.936	0.81 (1.70)	0.87 (3.88)	1.11 (1.97)	0.395	1.27 (2.23)	1.12 (2.09)	1.40 (2.34)	0.601
Psychological disability	1.13 (2.09)	1.36 (2.19)	1.35 (2.28)	0.443	0.25 (1.01)	0.58 (1.45)	0.74 (1.77)	0.199	0.78 (1.80)	0.98 (1.90)	1.05 (2.06)	0.223
Social disability	0.49 (1.29)	0.23 (1.01)	0.44 (1.39)	0.348	0.04 (0.27)	0.2 (0.85)	0.21 (0.83)	0.267	0.31 (1.04)	0.22 (0.93)	0.33 (1.15)	0.708
Handicap	0.64 (1.59)	0.78 (1.45)	1.07 (2.22)	0.387	**0.13 (0.54)**^**a**^	**0.61 (2.15)**^**b**^	**0.57 (1.52)**^**b**^	0.043*	0.44 (1.31)	0.7 (1.45)	0.83 1.93)	0.060

*G* Generalized, *L* Localized, *ND* No Disease

# Kruskal-Wallis test. Significant differences identified in bold (*p < 0.05)

OHIP-14 - Oral Health Impact Profile-14; SD – standard deviation

**Table 3:** Oral Health Impact Profile (OHIP-14) scores, total and for each domain, presented as mean and standard deviation (SD), according to gender and periodontitis severity

	Women (***n*** = 320)					Men (***n*** = 272)					Overall (***N*** = 592)				
Stage	0	1	2	3	P-value #	0	1	2	3	P-value #	0	1	2	3	P-value #
**OHIP-14. mean (SD)**	9.0 (11.9)	6.3 (8.9)	8.6 (10.4)	12.8 (13.2)	0.101	4.3 (6.5)	6.3 (9.0)	5.6 (7.4)	7.0 (10.3)	0.502	7.2 (10.3)	6.3 (8.9)	7.1 (9.2)	9.9 (12.2)	0.225
**OHIP-14 domain. Mean (SD)**															
Functional limitation	1.2 (2.2)	0.8 (1.6)	1.4 (1.9)	1.3 (2.1)	**0.027**	0.7 (1.4)	1.3 (2.1)	0.6 (1.2)	1.0 (1.7)	0.216	1.0 (2.0)	1.0 (1.9)	1.0 (1.6)	1.2 (1.9)	0.466
Physical pain	2.4 (2.7)	1.9 (2.5)	2.2 (2.5)	3.4 (2.9)	0.410	1.8 (2.3)	1.7 (2.3)	2.0 (2.5)	2.0 (2.5)	0.704	2.2 (2.6)	1.8 (2.4)	2.1 (2.5)	2.7 (2.7)	0.397
Psychological discomfort	1.6 (2.6)	1.1 (2.2)	1.5 (2.3)	2.3 (2.9)	0.293	0.5 (1.7)	1.0 (2.3)	1.0 (1.8)	1.0 (2.1)	0.126	1.2 (2.3)	1.0 (2.2)	1.2 (2.1)	1.6 (2.6)	0.190
Physical disability	1.6 (2.5)	1.0 (1.9)	1. 4 (2.7)	1.1 (2.1)	0.404	0.8 (1.7)	1.0 (1.8)	0.9 (1.8)	1.1 (2.2)	0.835	1.3 (2.2)	1.0 (1.9)	1.2 (2.2)	1.6 (2.5)	0.715
Psychological disability	1.1 (2.1)	1.0 (2.0)	1.1 (2.0)	1.8 (2.5)	0.765	0.3 (1.0)	0.7 (1.4)	0.5 (1.3)	0.9 (2.0)	0.266	0.8 (1.8)	0.9 (1.8)	0.8 (1.7)	1.3 (2.3)	0.725
Social disability	0.5 (1.3)	0.0 (0.2)	0.4 (1.2)	0.6 (1.6)	0.112	0.0 (0.3)	0.2 (0.8)	0.2 (0.5)	0.3 (1.0)	0.300	0.3 (1.0)	0.1 (0.6)	0.3 (1.0)	0.4 (1.3)	0.261
Handicap	0.6 (1.6)	0.5 (1.4)	0.7 (1.8)	1.4 (2.3)	0.946	0.1 (0.5)	0.5 (1.1)	0.4 (1.1)	0.8 (1.9)	0.101	0.4 (1.3)	0.3 (1.3)	0.5 (1.5)	1.1 (2.1)	0.493

0 – Healthy; 1 – Stage I, Mild Periodontitis; 2 – Stage II, Moderate Periodontitis; 3 – Stage III, Severe/Advanced Periodontitis; OHIP-14 - Oral Health Impact Profile-14; SD – standard deviation

# Kruskal-Wallis test. Significant differences identified in bold (*p < 0.05)

### OHIP-14 and covariates impact

Spearman’s rank-order correlation coefficient was used to investigate the possible correlation between total and each domain of OHIP-14 with SXI-5 total, PSS-10 total, number of missing teeth, mean probing depth (PD), mean clinical attachment loss (CAL), mean gingival recession (Rec) and mean bleeding on probing (BoP) (Table 4). SXI-5 total and the number of missing teeth had small positive significant correlations with OHIP-14 and each domain scores.

**Table 4:** Correlation of OHIP-14 total and domain scores with Summated Xerostomia Inventory-5 (SXI-5), Perceived Stress Scale-10 (PSS-10) scores, number of missing teeth, mean PD, mean CAL, mean Rec and Mean BoP

	OHIP-14							
	Total	Functional limitation	Physical pain	Psychological discomfort	Physical disability	Psychological disability	Social disability	Handicap
SXI-5 Total	0.281**	0.246**	0.213**	0.185**	0.207**	0.218**	0.143**	0.192**
PSS-10 Total	0.083*	0.008	0.019	0.093*	0.017	0.114***	0.053	0.115**
Number of missing teeth	0.184**	0.165**	0.105**	0.158**	0.182**	0.135**	0.091**	0.125**
Mean PD	0.126**	0.043	0.097*	0.118**	0.092*	0.095*	0.068	0.084*
Mean CAL	0.162**	0.089*	0.090*	0.140**	0.131**	0.117**	0.077	0.124**
Mean Rec	0.136**	0.085*	0.066	0.115**	0.106**	0.095*	0.054	0.115**
Mean BoP	0.110**	−0.015	0.105*	0.109**	0.071	0.071	0.060	0.090*

Overall trend across OHIP-14 scores, total and for each domain, assessed by Spearman’s rank correlation coefficient (rho). Significant correlations identified in bold (*p < 0.05, **p < 0.01)

*Mean BoP* Mean bleeding of probing, *Mean CAL* Mean clinical attachment loss, *Mean PD* Mean probing depth, *Mean Rec* Mean gingival recession, *OHIP-14* Oral Health Impact Profile-14, *PSS-10* Perceived Stress Scale-10, *SXI-5* Summated Xerostomia Inventory-5

To investigate which variables impacted the OHIP-14 total score, a multiple linear regression analysis was conducted (Table 5). Afterwards, age, the SXI-5 total, the PSS-10 total, missing teeth and mean PD significantly contributed to the OHIP-14 score. Notably, the factors that most contributed for the OHIP-14 score were mean PD (B = 1.56, 95% CI: 0.62–2.51) and SXI-5 (B = 1.20, 95% CI: 0.77–1.63). Conversely, age was a negative contributor (B = − 0.24, 95% CI: − 0.36, − 0.97).

**Table 5:** Multiple linear regression describing the influence of continuous variables on the total OHIP-14 score, with regression coefficients (B) and correspondent 95% confidence intervals (95% CI)

	OHIP-14 Total Score	
	B	***p***-value
**Age**	−0.24 (− 0.36, − 0.97)	< 0.001***
**SXI-5 Total**	1.20 (0.77–1.63)	< 0.001***
**PSS-10 Total**	0.35 (0.26–0.45)	< 0.001***
**Missing teeth**	0.24 (0.13–0.35)	< 0.001***
**Mean PD**	1.56 (0.62–2.51)	0.001**

**p < 0.01 and ***p < 0.001. R^2^ = 0.51

*OHIP-14* Oral Health Impact Profile-14, *PSS-10* Perceived Stress Scale-10, *SXI-5* Summated Xerostomia Inventory-5

A multivariate logistic regression, with Oral Health Impact Profile (OHIP-14) dichotomised into less affected vs frequently affected, was also carried out (Table 6). Overall, age, SXI-5 total, PSS-10 total, gender and mean CAL were significant for the model. While age has a negative effect (OR = 0.96, 95% CI: 0.93–0.99), being male (OR = 1.44, 95% CI: 1.00–2.06), SXI-5 total (OR = 1.28, 95% CI: 1.14–1.43) and mean CAL (OR = 1.25, 95% CI: 1.10–1.41) increase the risk towards a poorer quality of life.

**Table 6:** Multivariate stepwise logistic regression, with Oral Health Impact Profile (OHIP-14) dichotomised into less affected vs frequently affected, with Odds ratio (OR) and correspondent 95% CI

	OHIP-14 Total Score	
	OR (95% CI)	***p***-value
**Age**	**0.96 (0.93–0.99)**	0.003**
**SXI-5 Total**	**1.28 (1.14–1.43)**	< 0.001***
**PSS-10 Total**	**1.03 (1.00–1.05)**	0.022*
**Gender**		
Female	**1**	**–**
Male	**1.44 (1.00–2.06)**	0.048*
**Mean CAL**	**1.25 (1.10–1.41)**	< 0.001***

*The model was statistically significant, χ2(5) = 71.041, p < 0.001, explained 15.1% (Nagelkerke R^2^) of the variance and correctly classified 63.3% of cases

*Mean CAL* Mean clinical attachment loss, *OHIP-14* Oral Health Impact Profile-14, *PSS-10* Perceived Stress Scale-10, *SXI-5* Summated Xerostomia Inventory-5

In both analyses, the SXI-5 and PSS-10 significantly influenced the OHRQoL perception (Tables 4 and 5).

## Discussion

In this study, we hypothesized that self-perceived xerostomia and stress can change OHRQoL perception, analysing simultaneously the periodontal status, number of missing teeth, clinical characteristics and other variables. To test this hypothesis, through a significant dataset from a representative elderly population, we developed multivariable regression analyses accounting for these variables. Our results confirmed that self-perceived xerostomia and stress are associated with OHRQoL. The number of missing teeth, gender, and mean PD and CAL had a meaningful association to predict OHRQoL.

These findings have wide implications. (1) Self-perceived xerostomia revealed to be influential on OHRQoL in a population of elders, with a similar magnitude to the extent of periodontitis. (2) Self-perceived stress has a mild influence on OHRQoL. (3) The number of missing teeth and age are important variables to the OHRQoL variation. (4) The periodontal status does not influence the OHRQoL, rather some clinical features do. (5) As a result, self-perceived xerostomia and stress are important variables towards the OHRQoL in elderly patients.

As previously debated [22], the results of this epidemiological study indicate a disturbing prevalence of periodontitis among this elderly population and affecting more men. Further, this population reported faulty oral hygiene habits in agreement with a previous national report [29]. These characteristics may explain the high number of missing teeth and, as a consequence, more than half of the population were denture wearers. Besides, this population reported high levels of xerostomia and stress with significant differences between gender.

In terms of potential variables on the OHRQoL perception, SXI-5 exhibited a meaningful effect, while the perception of stress was meaningful but with mild impact. Comprehensively, the higher the perception of xerostomia and stress factors the higher odds of poorer OHRQoL. These results are in line with recent literature where perceived chronic stress impacted the perception of dry mouth and quality of life [18, 21, 30].

Two recent systematic reviews asserted that periodontal diseases might have an impact on OHRQoL [9, 31]. Though none of them had centred exclusively on elder populations, higher disease severity leads to a greater negative impact on OHRQoL. The results of this study agree with the latter since greater PD and CAL values led to worse OHRQoL, although neither the periodontal condition nor the extent of the disease had an influence on the quality of life perception. Besides, the number of missing teeth was a significant factor and complies with a recent systematic review in which retention of teeth is associated with better OHRQoL [32]. Apparently, patients esteem much more the number of missing teeth for their quality of life than having periodontitis or its extension and confirms what has already been reported [13, 14, 33]. The number of missing is a characteristic that patients often recognize, though with periodontitis this perception is scarce [22, 34]. Recently, we have validated a brief perception questionnaire for periodontal diseases [34], and it will be interesting to associate the perception levels towards periodontitis with the OHRQoL impact.

The association between OHRQoL and gender has been addressed in many investigations. In our report, women perceived poorer OHRQoL than men similarly to what has been found before [18, 33, 35, 36], although there are contrary results showing that men report poorer OHRQoL [37], or no difference whatsoever in the perception between men and women [13]. Still, we demonstrate that men have a higher risk of a frequently affected quality of life than women, possibly because of the overall poorer periodontal condition reported.

### Strengths and limitations

The results provided by our investigation have some notable strengths, as previously proposed [9]. We employed a full-mouth protocol with circumferential inspection, ensuring precise estimation of the prevalence and extent of periodontitis [38]. Also, we used the new AAP/EFP joint case definition with an up-to-date diagnosis with PD and CAL combination analysis. Besides, these results are representative and with adequate sample size calculation, stratified for each health centre. Further, we have included a periodontally healthy control group and possible factors.

However, there are limitations worth to mention in our study. Although we have included the number of missing teeth we could not account for occlusal pairs that have proven impact on OHRQoL [32]. The lack of information related to dentinal sensitivity as a result of gingival recession was also a limitation and shall be considered in the future. Further, the control group was derived from the same sample which can be seen as a possible shortcoming. Too, we employed the OHIP-14 which is more focused on the impact of pain on the patient’s psychological and behavioural traits, while the Geriatric Oral Health Assessment Index (GOHAI) tool is more suitable to examine functional limitations in relation to pain [39, 40] and ought to be considered in further investigations.

## Conclusions

We demonstrate that self-perceived xerostomia and stress are significant variables for the total score of OHRQoL in elderly patients, that is, they worsen the quality of life. Also, the significance was maintained even when analyzed simultaneously with the periodontal status, the number of missing teeth, periodontal clinical measures and oral hygiene habits. Future studies should consider these parameters when investigating the impact of periodontitis in quality of life.

## Data Availability

Due to legal arrangements with governmental institutions, we are not authorized to disclose data.

## Abbreviations

BoP: Mean bleeding on probing
CAL: Clinical Attachment Loss
CI: Confidence Interval
EMDC: Egas Moniz Dental Clinic
OHIP-14: Oral Health Impact Profile-14
OHRQoL: Oral Health Related Quality of Life
OR: Odds Ratio
PD: Pocket Depth
PROs: Patient-reported outcomes
PSS-10: Perceived Stress Scale-10
Rec: Mean gingival recession
SD: Standard Deviation
SoPHiAS: Study of Periodontal Health in Almada-Seixal
STROBE: STrengthening the Reporting of OBservational studies in Epidemiology
SXI-5: Summated Xerostomia Inventory-5

## References

[1] Baumhauer J (2017). Home-to-home time — measuring what matters to patients and payers. N Engl J Med.

[2] Johnston BC, Patrick DL, Busse JW, Schünemann HJ, Agarwal A, Guyatt GH (2013). Patient-reported outcomes in meta-analyses--part 1: assessing risk of bias and combining outcomes. Health Qual Life Outcomes.

[3] World Health Organisation (2015). World Report on Ageing and Health.

[4] Albandar JM (2002). Global risk factors and risk indicators for periodontal diseases. J Periodontol 2000.

[5] Dye BA (2012). Global periodontal disease epidemiology. Periodontol 2000.

[6] Persson GR (2017). Dental geriatrics and periodontitis. Periodontol 2000.

[7] Shanbhag S, Dahiya M, Croucher R (2012). The impact of periodontal therapy on oral health-related quality of life in adults: a systematic review. J Clin Periodontol.

[8] Al-Harthi L, Cullinan M, Leichter J, Thomson W (2013). The impact of periodontitis on oral health-related quality of life: a review of the evidence from observational studies. Aust Dent J.

[9] Buset SL, Walter C, Friedmann A, Weiger R, Borgnakke WS, Zitzmann NU (2016). Are periodontal diseases really silent? A systematic review of their effect on quality of life. J Clin Periodontol.

[10] Foltyn P (2015). Ageing, dementia and oral health. Aust Dent J.

[11] Mariño R, Albala C, Sanchez H, Cea X, Fuentes A (2015). Prevalence of diseases and conditions which impact on oral health and oral health self-care among older chilean. J Aging Health.

[12] Botelho J, Machado V, Proença L, Bellini DH, Chambrone L, Alcoforado G (2020). The impact of nonsurgical periodontal treatment on oral health-related quality of life: a systematic review and meta-analysis. Clin Oral Investig.

[13] Kato T, Abrahamsson I, Wide U, Hakeberg M (2018). Periodontal disease among older people and its impact on oral health-related quality of life. Gerodontology..

[14] da Mata C, Allen PF, McKenna GJ, Hayes M, Kashan A. The relationship between oral-health-related quality of life and general health in an elderly population: a cross-sectional study. Gerodontology. 2019;36(1):71–7. 10.1111/ger.12384. 10.1111/ger.12384 30536976

[15] Botelho J, Machado V, Mascarenhas P, Rua J, Alves R, Cavacas MA (2018). Stress, salivary cortisol and periodontitis: A systematic review and meta-analysis of observational studies. Arch Oral Biol.

[16] Tay KJ, Yap AUJ, Wong JCM, Tan KBC, Allen PF (2019). Associations between symptoms of temporomandibular disorders, quality of life and psychological states in Asian military personnel. J Oral Rehabil.

[17] Niklander S, Veas L, Barrera C, Fuentes F, Chiappini G, Marshall M (2017). Risk factors, hyposalivation and impact of xerostomia on oral health-related quality of life. Braz Oral Res.

[18] Thomson WM, Ibrahim H, Lyons KM, Foster Page LA, Hanlin SM (2019). Personality, xerostomia and OHRQoL among 35–54-year-olds. Acta Odontol Scand.

[19] Gerdin E, Einarson S, Jonsson M, Aronsson K, Johansson I (2005). Impact of dry mouth conditions on oral health-related quality of life in older people. Gerodontology.

[20] Johansson AK, Johansson A, Unell L, Ekbäck G, Ordell S, Carlsson GE. Self-reported dry mouth in 50- to 80-year-old Swedes: Longitudinal and crosssectional population studies. J Oral Rehabil. 2020;47(2):246-54. 10.1111/joor.12878. 10.1111/joor.12878 31444791

[21] Bulthuis MS, Jan Jager DH, Brand HS (2018). Relationship among perceived stress, xerostomia, and salivary flow rate in patients visiting a saliva clinic. Clin Oral Investig.

[22] Botelho J, Machado V, Proença L, Alves R, Cavacas MA, Amaro L (2019). Study of periodontal health in Almada-Seixal (SoPHiAS): a cross-sectional study in the Lisbon metropolitan area. Sci Rep.

[23] von Elm E, Altman DG, Egger M, Pocock SJ, Gøtzsche PC, Vandenbroucke JP (2014). The strengthening the reporting of observational studies in epidemiology (STROBE) statement: guidelines for reporting observational studies. Int J Surg.

[24] Tonetti MS, Greenwell H, Kornman KS (2018). Staging and grading of periodontitis: framework and proposal of a new classification and case definition. J Periodontol.

[25] Afonso A, Silva I, Meneses R, Frias-Bulhosa J (2017). Oral health-related quality of life: Portuguese linguistic and cultural adaptation of Ohip-14. Psicol Saúde Doença.

[26] de Amaral JP AR, Marques DN d S, Thomson WM, Vinagre ARR, da Mata ADSP (2018). Validity and reliability of a Portuguese version of the summated Xerostomia Inventory-5. Gerodontology..

[27] Trigo M, Canudo N, Branco F, Silva D (2010). Estudo das propriedades psicométricas do Perceived Stress Scale. Psychologica..

[28] Cohen S, Kamarck T, Mermelstein R (1983). A global measure of perceived stress. J Health Soc Behav.

[29] Santos J, Antunes L, Namorado S, Kislaya I, João Santos A, Rodrigues AP (2019). Oral hygiene habits in Portugal: results from the first health examination survey (INSEF 2015). Acta Odontol Scand.

[30] Echeverria MS, Wünsch IS, Langlois CO, Cascaes AM, Ribeiro Silva AE (2019). Oral health-related quality of life in older adults—longitudinal study. Gerodontology..

[31] Ferreira MC, Dias-Pereira AC, Branco-de-Almeida LS, Martins CC, Paiva SM (2017). Impact of periodontal disease on quality of life: a systematic review. J Periodontal Res.

[32] Tan H, Peres KG, Peres MA (2016). Retention of teeth and oral health-related quality of life. J Dent Res.

[33] Masood M, Newton T, Bakri NN, Khalid T, Masood Y (2017). The relationship between oral health and oral health related quality of life among elderly people in United Kingdom. J Dent.

[34] Machado V, Botelho J, Ramos C, Proença L, Alves R, Cavacas MA, et al. Psychometric properties of the Brief Illness Perception Questionnaire (Brief-IPQ) in Periodontal Diseases. J Clin Periodontol. 2019. 10.1111/jcpe.13186. 10.1111/jcpe.13186 31446628

[35] Cornejo M, Pérez G, de Lima KC, Casals-Peidro E, Borrell C (2013). Oral health-related quality of life in institutionalized elderly in (Spain) Barcelona. Med Oral Patol Oral Cir Bucal.

[36] Ulinski KGB, Do Nascimento MA, Lima AMC, Benetti AR, Poli-Frederico RC, Fernandes KBP (2013). Factors related to oral health-related quality of life of independent Brazilian elderly. Int J Dent.

[37] Pattussi MP, Peres KG, Boing AF, Peres MA, Da Costa JSD (2010). Self-rated oral health and associated factors in Brazilian elders. Community Dent Oral Epidemiol.

[38] Botelho J, Machado V, Proença L, Mendes JJ (2020). The 2018 periodontitis case definition improves accuracy performance of full-mouth partial diagnostic protocols. Sci Rep.

[39] Adamo D, Pecoraro G, Fortuna G, Amato M, Marenzi G, Aria M (2020). Assessment of oral health-related quality of life, measured by OHIP-14 and GOHAI, and psychological profiling in burning mouth syndrome: a case-control clinical study. J Oral Rehabil.

[40] Lee KH, Jung ES, Choi YY (2020). Association of oral health and activities of daily living with cognitive impairment. Gerodontology..

